# Theorising post-truth in the COVID era

**DOI:** 10.1007/s10833-022-09457-3

**Published:** 2022-04-19

**Authors:** David Nally

**Affiliations:** University of Newcastle, Callaghan, Australia

**Keywords:** Post-Truth, Education, COVID-19, Post-pandemic recovery, Entrepreneurship

## Abstract

The focus of this article is on the impacts of COVID-19 related manifestations of post-truth in educational settings in Australia. Within this context, there has been a reorientation of how wellbeing and academic achievement within schools reflect on broader trends within the general public, at local, state and national scales. Individual and communal experiences of adversity have been significantly impacted by phenomena associated with post-truth, particularly misinformation, a climate of anti-intellectualism, as well as fragmented socio-cultural cohesion. In the first section I explore these trends by providing an overview of how post-truth has been construed in Australian contexts, before moving to consider how engagements with post-truth have been shaped by the pandemic. Second, I analyse the close link between educational concerns that emerged from the pandemic era, and the circumstances that have supported the emergence of post-truth. Particular attention will be paid to debates over ‘learning loss’ and the place of teachers within Australian communities as a fulcrum for generating cultural capital and social cohesion. In the final section I consider what lessons these experiences have for education, as a way of cultivating learning communities that are oriented towards generating critical and digital literacy skills

## Introduction

This essay describes the impacts of COVID-19 related manifestations of post-truth in educational settings. Here, I focus on specifically on Australia, where there has been a reorientation of how wellbeing and academic achievement within schools reflect on broader trends within the general public, at local, state and national scales. Individual and communal experiences of adversity have been significantly impacted by phenomena associated with post-truth, particularly misinformation, a climate of anti-intellectualism, as well as fragmented socio-cultural cohesion. These concerns are not unique to Australia; throughout the essay I suggest how the challenges facing Australia apply more globally.

The ongoing effects of COVID-19 on societies mirror its impacts on education; advantages and disadvantages embedded in economic and social inequalities have been more publicly visible, as have cultural and political divisions. Further, there has been a glossing over of truth-value in public discourse, with an inclination instead towards either responses that are health-driven and those that are motivated by economic gain or solidifying an elite status within political or business hierarchies. The result has been, according to a McCrindle report on projections for Australia in 2031, a de-centralisation of the world of work; a reflection on the use, availability and division of public and private spaces; as well as calls in many Western countries for re-thinking the design of capital cities, smaller centres, and rural towns (McCrindle, Renton & Leung, 2021). Across the member countries of the Organisation for Economic and Cultural Development (OECD), as many populations come to terms with how to adapt to a pandemic as it mutates to being endemic to specific regions, there is a fracturing of what is deemed essential and valued in individuals, local community and governmental scales, as well as in relation to business, cultural, and social welfare groups (Azevedo et al., 2021; Hanushek & Woessmann, 2020; Sacks, Bayles, Taggart, & Noble, 2021; Also see Hammerstein et al. (2021) for a meta-analysis of literature on the topic). This difference in perspective stems from what is deemed as essential for recovering from the ongoing impacts of COVID-19: If the pandemic is considered to stem from a crisis in public health, then there must be considerations of the ramifications for environmental, ethical, personal and societal concerns.

Numerous reports documenting the educational experiences resulting from COVID-19 during 2019–2021 have focussed on issues such as learning loss, wellbeing concerns (especially psychological), the inequity faced by students in a purely home learning environment during lockdowns, linguistic and technological barriers to accessing support and materials, as well as the discrepancy in achievement based on motivation of students, their parents, and educators (See Merga et al., 2021; Smith et al., 2021). In this essay, I argue that these conditions in a schooling context reflect a broader trend of post-truth trickling into public discourse about education. As a case in point, I examine the degree to which post-truth has developed in an Australian context in correspondence with interpretations of the concept. I will then move to consider how COVID-19 exacerbated several pre-pandemic trends in Australia which are relevant to more global contexts: especially forms of inequality, exclusion, and forms of political messaging that are aimed at obfuscating truth. In the second section, I briefly analyse how closely educational concerns that emerged from the pandemic are linked with the structures that have supported the emergence of post-truth. Particular attention will be paid to debates over 'learning loss,' school closures, and the place of teachers within Australian communities as a fulcrum for generating cultural capital. Finally, the last section concludes with a consideration of what lessons these experiences have for future teaching practice, as a way of cultivating learning communities that are oriented towards building cultural capital, resilience, as well as critical and digital literacy skills.

## You can’t handle the [post-]truth!

In a pre-COVID-19 Australia, the majority of scholarship concerned with post-truth construed it as the denial of—or the refusal to recognise—knowledge that underpins forms of socio-political exclusion. The most cited Australian examples include the denial of sexual abuse and misogyny (Burke & Carolissen, 2018), genocides against Australian Indigenous (Gudonis & Jones, 2020), and the Holocaust (Lipstadt & Koval, 2019; Malpas, 1992). These case studies catalysed Mattias Gudonis and Benjamin Jones’ conception of post-truth, which attempts to reconcile several older definitions to reflect on what intentions motivate denials of reality and spread of misinformation:

Post-truth history is the communication of false information on a historical phenomenon that appeals to emotion and personal belief, where both the purveyor and recipient are indifferent to historicity and contemptuous of expert opinion that contradicts it, and where the underlying objective is ideological, especially in support of a collective identity or a political programme. Gudonis & Jones (2020: 8)

This definition constitutes a cross-section about how post-truth has developed as a concept over the past three decades. While Eric Malpas’ (1992) definition was preoccupied with how historical reality can be denied, the definitions of Steve Tesich (1992), Ralph Keyes (2004) and Steve Fuller (2018) pertained to authority figures (in government, business, and culture) who concealed and manipulated facts for self-protection and interest that would otherwise be in the public interest to be reported accurately. Where the ‘collective identity or a political program’ (Gudonis & Jones, 2020) might be elaborated upon then, is in relation to a trend of curtailing resistance to authority figures, by framing decisions in terms of public security. It can therefore be argued that the emphasis on public safety during the pandemic is the latest manifestation of this characteristic, on the basis that it minimises resistant political discourse as well as projecting an image of political dominance.

One significant consequence of these developments mirrors a behavioural observation made by Michel Foucault (1977: 135–142), that the public has the potential to be transformed into ‘docile bodies.’ This term refers to the capacity of individuals to internalise messages they receive from figures in authority, and over time grow to independently replicate them. In the case of COVID-19 this point is borne out by the popularity gained by political figures, especially when security and health have been positioned as a core part of ‘political program[s]’ and have been wedded to the public interest as part of responding to COVID-19.

This brief overview of discussions about post-truth attests that it refers, at least in part, to the process whereby political narratives work to preserve images of strength and provision of safety. Under these circumstances, prominent individuals, institutions, and political parties consolidate their supporter base at the same time as political divides become more clearly pronounced (Fuller, 2018; McIntyre, 2017; Tesich, 1992). Such trends are epitomised in several countries’ constituencies casting doubt over the legitimacy of their democratic forms of government to govern in the national interest (see for instance: Bleakley, 2018). Commonly cited case studies include: U.S.A. Politics (Davis, 2017; Fuller, 2018; Kalpokas, 2019; McIntyre, 2018); The Philippines’ Rodrigo Duterte’s policies (Ramirez, 2020; Tapsell, 2017), Brexit and U.K. politics (Davis, 2017; Kalpokas, 2019; Marshall & Drieschova, 2020; McIntyre, 2018), Putin and Russia (Kalpokas, 2019; Pomerantzev, 2016, 2019), Brazil’s Jair Bolsonaro (Consentino, 2020)).

These points have been borne out in the last decade in Australia by way of successive Federal governments from across the political spectrum adopting an inertia-driven approach to climate change (Beeson, 2021; Blackall, 2017; Connor, 2014; Grafton, 2020; Speck, 2010), challenging and directly interfering with the independence of academic research grants (Bradby, 2018; Walsh, 2022), as well as defunding of the arts, public journalism, and sciences where they could be construed as undermining a political party’s attempts to continue institutional legacies of their political predecessors (Tingle, 2014, 2016, 2019). According to John Keane, these developments have underpinned a global phenomenon of ‘new populism,’ which begins as ‘post-truth games’ being deployed by populist politicians that sow disillusionment with democratic institutions, as well as dismissing or ignoring public concerns if they did not align with their political agenda. Thus, what can be added to Gudonis and Jones’ definition is that post-truth appears to also be associated with the means by which public mistrust grows in apparatuses of government as a result of equating the responsibilities and legitimacy of offices with the individuals and parties who occupy them. These factors suggest why the spread of COVID-19 has resulted in post-truth being mentioned frequently in tandem with misinformation, denialism, and consequences of global inequalities.

In the wake of COVID-19, there has been a shift toward connecting personal circumstances with the health of an entire society (see for example: Harsin, 2020; Kwok et al., 2021; Parmet & Paul, 2020; Peters et al., 2020; Prasad, 2021; Valladares, 2021). As such, the problems posed by post-truth have been brought to public attention because personal welfare has been explicitly linked with education about—and public communication of—medical knowledge. Thus, I propose that it is more appropriate to define the concept as constituting three layers:

1. *Problematic epistemologies*, where emotional reactions are used as a starting point for cultivating an empirical understanding of the world. In this layer, present circumstances are cast as the product of a historical narrative of decline, as problems emerge.
2. *Behaviours that work to challenge norms which are based on cultural traditions, technical and academic expertise*. These include how forms of active citizenship can be expressed as part of organising groups that represent interests, businesses and communities.
3. *Factors which moderate how people engage with cultural, economic and political spaces*. These include technologies that allow access to digital spaces, in addition to forms of socio-economic inequality (such as divisions between classes, castes, and first–second-third world nations).

## Spreading post-truth trends in education

In the wake of the extensive disruption wrought by the pandemic, there has been a growing tendency away from simplifying post-truth to intentionally crafted misinformation and its impacts. Instead, there is a movement towards creating definitions that draw a conceptual thread between forms of educational, linguistic, cultural, and economic inequalities and their catalyst: what Fuller has labelled as ‘power games’ in political rhetoric that downplay forms of social divisions (2018: 2). Framing COVID-19 responses within the layers of post-truth outlined at the end of the first section accounts for how problematic epistemologies and divisive politics contributed significantly to viral spread.

In this sense, the emergence of post-truth accounts for the reactions to COVID-19, as well as attesting to how awareness of both phenomena can potentially be addressed by developing critical and political literacy amongst student cohorts as a starting point to address ‘learning loss,’ which is a factor discussed below. The case for utilising layers of post-truth in addressing the challenges posed by COVID-19 is reinforced by a report written by Natalie Brown et al. (2020: 5, 9) which found that by April during the first waves of COVID-19 in Australia, 46% of students fit within degrees of vulnerability pre-pandemic, or were newly vulnerable during the pandemic. Further, Dawson et al. cautioned that the usual need for remedial instruction would be compounded by the pre-pandemic difficulties involved in supporting students to ‘attain sufficient competence … in NAPLAN level II and PISA level I,’ in line with the Matthew effect (rich get richer; poor get poorer) (2020: 10).

For these considerations to be implemented, educators might realistically view part of their role as being a conduit for promoting social cohesion that consequently facilitates a recovery from the pandemic’s impacts. The need for such an approach is justified on educational as well as economic bases; several studies in Australia correlate with international findings in asserting that economic disadvantage and educational engagement during adolescence determine degrees of digital literacy, and in turn, workplaces’ adaptivity in a time of crisis (Black & Walsh, 2019; Hanushek & Woessmann, 2020; Sacks et al., 2021).

These ongoing effects about the educational impact of COVID-19 conjoined with economics have been the subject of much discussion. According to Per Engzell, Arun Frey and Mark Verhagen’s Dutch study (2021), the national learning effect size decreased 0.08 (or, 3% learning in a year), but for students who had parents with less education there was a decline of as much as 60%. Klaus Zierer used this study, along with others based in Switzerland, the USA, Belgium and Germany, to comment that based on 4.5 million students’ data, it is as yet unknown about what impacts school closures will have on student cohorts. Disadvantaged students however, have been most immediately affected however and future policy will need to address degrees of remedial learning with an ethic of ‘social justice’ (2021: 11). A third contribution that has been widely cited in relation to learning loss is John Hattie’s commentary that the impact of COVID-19 would likely mirror the aftermath of the 2003 Hurricane Katrina in the USA, or Christchurch’s earthquake in New Zealand during 2011. In these cases, students’ end of course results reflected a significant capacity to rebound from their losses during a 2–3 year period, in line with how effective communities recovered from natural disasters (2020).

Although these three recent studies examine experiences from quite different education systems, they do reflect that there must be an initial assumption that learning gaps will exist, and that digital media and remote learning offer opportunities in individualised remediation and instruction. These technological approaches need, however, to be combined with methods that encourage attendance and appropriate staffing allowances that both enable and compensate for the additional hours (Radinger & Boeskins, 2021). These considerations also need to mitigate substantial concerns associated with remote education, namely the preparedness in responsibilities for teachers, students, and their communities (Riddle, 2021); developing and sustaining student motivation (offsetting absenteeism, non-submission of work); as well as access to appropriate internet connections and digital technology (Gouedard et al., 2020).

In addressing the challenges of COVID-19, there is an opportunity to address an embedded, longer-term narrative of decline about education in Australia. Arguably, it might be extrapolated that this factor underpins the development of a post-truth political narrative that was earlier outlined in the first section as Layer 1. Pre-pandemic, declining PISA rankings (see Schleicher, 2019: 10) were leveraged by State and National Australian Governments to justify the need for reforms toward decentralising decision making from regional offices to principals, with the stated intention of reworking policies as needs-based as well as an emphasis on configuring teaching quality standards being used as a gauge for how wage increases and career progression could be awarded (Call, 2018; Crawford, 2020). These changes have been accompanied by an emphasis on accountability about teacher performance. This trend lies in contrast with the absence of explicit planning that involves cultivating a supportive culture amongst staff, let alone one that engages with the communities that feed into schools’ catchments (Luke et al., 2018; Ladwig, 2019). In this way, the culture promoted by this discourse is one that discourages initiative and autonomy. Echoing Foucault (1977), it encourages a self-regulated conformity amongst educators to what is expected of their role/s within a hierarchy, moreso than taking students’ needs as a starting point for developing a pedagogical relationship between teachers and students.

## Where do we go now? some suggestions for education

To address the challenges of COVID-19 and post-truth, the evidence surveyed in this brief essay indicates that there needs to be a balance of teaching to encourage a link between personal self-efficacy as being more effectively cultivated as part of a collective, a sense of connectedness between community and industry groups, as well as with a school’s physical environment. New South Wales—where I live—has the unenviable reputation of being the source of all three waves of COVID-19 in Australia. The spread here—as with a global basis—had an almost thrombotic effect; the wide range of divisions in Australian societies and education became more clear (Ainley et al., 2022; Seymour et al., 2020). In Sydney, the capital, it was as if one city became three during lockdowns:

- Manufacturing and trade-based economies of the central-west and south-west played host to dramatic increases in case numbers;
- Central and the north-west suburbs had somewhat lower case numbers depending on population densities and the ability to remain at home due to working remotely;
- Eastern suburbs meanwhile were characterised by initially high case numbers followed by sporadic outbreaks, in addition to a much less harsh treatment by police by comparison to everywhere else in the Sydney region. (For more on this, see Mude, et al. 2021).

These fractures were echoed by other divisions; in remote rural areas such as Wilcannia in New South Wales, there was a divide between Indigenous and non-Indigenous populations in economic terms as well as to access to vaccinations (Green, 2021; Menzel, 2021). As such, a history of mistrust in government institutions build on colonialism’s ongoing impact in this country underpinned an attitude of vaccine hesitation. A linguistic inequality was also highlighted, by way of official health and safety guidelines being available exclusively in English for quite some time before being translated in mid-2021. Moreover, a gendered inequality was manifested in a silent epidemic of domestic violence, particularly during the social isolation of lockdown (Boxall, et al., 2020; Carrington et al., 2020, Carrington et al., 2021), and a larger proportion of jobs that were lost were in female-dominated industries (Churchill, 2021; Craig & Churchill, 2021).

Educational approaches to a recovery from COVID-19 might therefore consider how knowledge of digital media and communication, educators’ professional development, and generation of cultural capital might be effectively integrated. A digital emphasis has been suggested as part of a much-cited examination of post-truth: Stephan Lewandowsky, Ulrich Ecker, and John Cook posit that conditions giving rise to it have been exacerbated in recent years due to ‘communication tools [that construct] … alternative epistemologies which defy conventional standards of evidence.’ (2017: 3) They go on to suggest a potential solution in the form of ‘technocognition’ (2017: 30, 37), where there is a shared priority between the general public, political groups, and digital business organisations for discouraging forms of misinformation. The way this notion could potentially work is how technocratic businesses (such as Alphabet and Amazon) might develop an architecture in their algorithms that does not necessarily encourage users to visit the most-visited products and sites. Instead, there could be filters embedded in searches to measure the reliability of sources, such as using metrics on citation numbers in a similar way to Google Scholar listings, as well as in relation to the author’s credibility. Although these factors are in digital businesses rather than education, awareness about them to provide foundations for teaching strategies might use planning principles from research conducted by Sam Sims and Harry Fletcher-Wood (2021; cf. Darling-Hammond et al., 2017). This meta-analysis of how professional development can be effective constitutes the widest ranging study on this topic in English, and argues that there must be a triangulation between students’, individual teachers’, and a school’s needs. Further, this study points out that there is a necessity for acknowledging that there will not be a universal uptake.

Perhaps the most prominent uptake of digital spaces is in the use of such technology to address how geographical restrictions on education might begin to be addressed, depending on the quality of—and access to—internet and digital technologies (Davies et al., 2021). It also allowed many institutions to deliver more sustainable educational experiences (preventing pollution from travel, saving time, and being more cost-effective) to students aged between 4 and 18 years as well as explore the extent to which distance and digital education strategies could be useful for mass-education. A notable finding across multiple countries has been that students’ engagement—rather than attendance—has been the most significant factor in their success in assessments (Ahshan, 2021; Schneider, 2021; Smith et al., 2021; Following Luke et al., 2018). The effectiveness of such accommodations however, significantly depended on students’ ability to attend video conferencing sessions. Another consideration is the degree to which these sessions conflicted with the flexibility required for access by educators, particularly those who had young children or elderly relatives to care for. While there was decreased transport time to and from work, there was a blurring of home-work distinction that contributed to fatigue.

Another potential antidote to the splintering of communities during COVID-19 might be found in streamlining students’ transition from education into the world of work, by teaching an ethos of self-regulation and entrepreneurship. According to Daniele Morselli’s analysis of how entrepreneurial education has developed - at a localised and continental level - this focus was linked, pre-pandemic, with numerous European countries’ policy intentions to leverage education as a springboard for economic growth, with the prospect of a higher standard of living going hand in hand with promoting ‘liberal ideals with personal freedom and citizenship at the centre, since the individual has freedom to change, develop, grow and adapt to contexts … and circumstances.’ (Morselli, 2019 : 7) This framing affords a solutions-based learning approach within authentic contexts, to embed an ethos of working towards a future recovery from a pandemic affected present. Examples of this in Australia are demonstrated by a high school initiative in Sydney, Up Rising, which has directly linked senior high school design students’ projects to local businesses, to generate both potential future career pathways as well as create a culture of appreciation for meaningful learning experiences that have a visible, real-world impact. Such authentic relationships are examples of clear alignment between student–teacher-school-community, which will address the narrative of decline in education, prevent various layers of post-truth becoming further entrenched, as well as mobilise a wide variety of interest groups in the recovery process from COVID-19 and regenerative opportunities for the natural environment (for a United Kingdom example in the Arts sector, see Hall and Thomson, 2019).

## Conclusion

In the third year of the COVID-19 pandemic, there is a need to consolidate what national policy papers such as *Through Growth to Achievement* called for in the wake of PISA results from 2018: that differentiation and school policies must be ‘adaptive and innovative’ based on incorporating a consistent approach to interpreting data, to make decisions based on equity and a needs-based ethos (2018, p. 112). This focus constitutes a step away from the accountability focussed, decentralised emphasis that has been cultivated over the last decade in response to PISA results, and opens the door for current and future policies determining schools’ success criteria to be more authentically focussed on student learning and needs-based funding models. A case in point can be recognised in the catering of micro-credentials for high school students as a way of students developing global capabilities, accelerating high performing students as well as future-proofing their learning.

A study by Heffernan, Magyar, Bright and Longmuir in the state of Victoria demonstrated that in the wake of the pandemic, there has been a widespread increase in respect for teachers and the value of skills and knowledge that is imparted within schools (2021). On the basis of this finding, learning-oriented partnerships between schools and their local communities might be re-established to develop cultural capital in a manner that allows for a re-engagement of disengaged students. Absenteeism of students appears to have been a global issue during lockdowns, where participation was digital rather than in-person, as well as dependent on teachers’ and students’ quality of technological access and socio-economic status.

What will hopefully begin to develop as a result of COVID-19 is the view that all members of a school community represent a part of an interest group, which have a human side with which they need to demonstrate empathy at some point. Through a post-truth lens however, there is a caution that this vision will be balanced with a tendency to further entrench hierarchies of accountability, while augmenting them with the digital innovations and surveillance that have taken place during the pandemic. This thinking should underpin any implementation of critical and political literacy, but in such a way authorities, teachers, parents, students and organisations that are part of a school community seek to establish themselves as part of a whole, rather than necessarily in friction with one another.

In turn, such an ethos works to combat the narrative of decline embedded in post-truth discourse, and is a starting point for balancing education-based responses with the need to pave the way for social, economic and health infrastructure recovery in the wake of COVID-19. These factors may well help to cultivate collective efficacy that will be essential in arresting the much discussed decline in student performance in NAPLAN and PISA (Riddle, 2021; Thomson et al., 2019). Finally, the current available data does not show a more complete picture of student learning throughout the pandemic. Workable statistics from student progress—as well as wellbeing data—will therefore only begin to emerge in results of school finishers at the conclusion of 2022. This consideration underlines the need for more studies to take place that focus on the factors that influence effective transition from remote learning back into schooling environments.
